# Comparative Evaluation of 5-*n*-Alkylresorcinol Extraction Conditions from Wheat Bran via Metabolite Profiling: Implications for Antiproliferative Activity

**DOI:** 10.3390/foods14122108

**Published:** 2025-06-16

**Authors:** Ronald Marentes-Culma, Ericsson Coy-Barrera

**Affiliations:** Bioorganic Chemistry Laboratory, Universidad Militar Nueva Granada, Cajicá 250247, Colombia; ronaldmarentes1994@gmail.com

**Keywords:** wheat bran, Alkylresorcinols, extraction, chemical profiles, Antiproliferative

## Abstract

Alkylresorcinols (ARs) are bioactive phenolic lipids with potential health-promoting properties; they are particularly abundant in wheat bran. This study aimed to compare the efficiency of eight extraction methods—including Soxhlet, ultrasound-assisted extraction (UAE), and overnight solvent-assisted maceration (OSAM) protocols—used in the removal of ARs from wheat bran, and to evaluate the association between AR composition and antiproliferative activity. A metabolite profiling approach using HPLC-DAD-MS identified 12 AR homologs differing in side-chain length and functional groups. Among the extraction strategies, the UA extraction, OSAM and Soxhlet-assisted with only acetone yielded the highest concentrations of bioactive ARs, particularly the C_17_–C_25_ homologs characteristic of cereals. Sparse partial least squares discriminant analysis (sPLS-DA) determined the discrimination of the different extraction methods, while variable importance scores revealed that AR homologs such as C_25_, C_19:1_, and C_23:Oxo_ were key to the distinguishment of the extraction methods. Antiproliferative assays against PC-3 prostate cancer cells (IC_50_ = 13.3–55.6 µg/mL) demonstrated that extracts rich in oxygenated ARs exhibited significantly higher antiproliferative effects than those dominated by saturated compounds. This finding suggests that both side-chain length and functionalization (e.g., keto groups) influence AR bioactivity. These findings suggest that extraction conditions can be optimized not only to enhance AR yield but also to enrich homologs with higher antiproliferative potential, providing foundations for exploring AR-enriched products derived from wheat bran.

## 1. Introduction

**Scheme 1:**
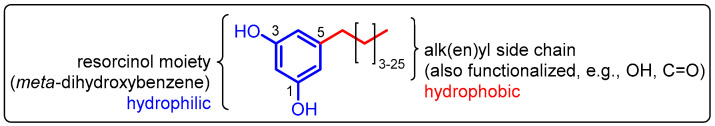
General structure of alkyresorcinols (AR), with labels for the resorcinol ring (hydrophilic moiety) and alkyl side chain (hydrophobic moiety).

Alkylresorcinols (ARs) comprise a group of chemical compounds structurally related to a *meta*-dihydroxy-substituted benzene (namely, resorcinol moiety) having an alkyl (or alkenyl) side chain at the C_5_ position [1]. These homologous compounds primarily differ in the length of the side chain—ranging from 5 to 27 carbon atoms—and in their degree of unsaturation and as to the presence of functional groups. Owing to their structural features, ARs exhibit amphipathic properties: the phenolic ring contributes to hydrophilicity, whereas the long aliphatic chain imparts hydrophobicity (Scheme 1) [1]. However, ARs exhibit lipophilicity, and are therefore classified as phenolic lipids, with their octanol/water partition coefficients (log P_o/w_) ranging from 7 to 10, depending on the length of the side chain [2].

Although various organisms can synthesize ARs, they are predominantly found in plants—especially in members of the Poaceae family [1]. High concentrations are typically observed in the outer layers of cereal grains such as wheat, rye, and triticale, particularly in the bran and the intermediate layers of the kernel [3]. However, there is also some evidence of their presence throughout the whole plant [4]. ARs have attracted considerable interest due to their reported biological activities, including antioxidant [5] and antimicrobial properties [6], as well as antiproliferative potential against cancer cells [7]. As such, their dietary intake has been associated with potential health benefits, supporting the consideration given them as biomarkers for whole-grain consumption and disease risk reduction [8].

Wheat bran, the outer layer of the wheat kernel separated as a by-product of the milling process, is one of the most nutritionally rich components of wheat grain. It is a popular source of dietary fiber, often added to baked goods, breakfast cereals, and other foods to increase fiber content, and it can be eaten directly as a supplement [9]. It also contains sufficient levels of vitamins and antioxidants, as well as various phytochemicals, including ARs [10]. These components confer prebiotic properties upon the wheat bran, and have been associated with improved gut health, glycemic control, and reduced risk of cardiovascular disease [11,12]. Among its bioactive compounds, the presence of ARs in the wheat bran—a highly accessible and low-cost source—further enhances its functional value, expanding its potential applications in food enrichment [13,14]. Despite its nutritional richness, wheat bran may also contain trace levels of heavy metals and environmental contaminants, which can accumulate in the outer kernel layers [15]. Therefore, extraction techniques that selectively recover antioxidant and bioactive components while minimizing co-extraction of potential toxicants are highly desirable for food and nutraceutical applications.

Recent studies have demonstrated that ARs exert anticancer effects, particularly against prostate cancer cells. Extracts rich in ARs have shown selective cytotoxicity towards PC-3 cells, inducing apoptosis and the inhibition of cell proliferation through mechanisms involving mitochondrial dysfunction, modulation of oxidative stress, and disruption of the cell cycle progression [16]. Importantly, the efficacy of these extracts appears to be highly dependent on their AR homolog composition. Differences in the side chain length and unsaturation significantly influence their bioavailability and potency, with shorter-chain ARs (e.g., C_13_–C_17_) exhibiting enhanced antiproliferative activity, likely due to increased lipophilicity and membrane affinity [17]. Thus, the extraction method not only affects total-AR yield but also determines the qualitative profile of ARs in the final extract, ultimately influencing its biological efficacy [18].

To harness the health-promoting properties of ARs, particularly for cytotoxic assays, it is essential to obtain extracts enriched in bioactive AR homologs. Extraction plays a pivotal role in removing these bioactive compounds from complex cereal matrices. Among the available approaches, organic solvent-based maceration—a traditional solid–liquid extraction method involving prolonged contact (typically 24 h)—is the method most commonly employed [19,20]. However, the efficiency of this method depends on multiple factors, such as the solvent’s ability to penetrate the matrix, its affinity for the solute (ARs), and the diffusion rate of ARs within the solvent phase [21]. Considering the amphipathic nature and lipophilicity of ARs, solvents of intermediate polarity, such as ethyl acetate and acetone, are generally effective [22]. Other (semi)automated methods for the removal of ARs from cereals, including those using high temperatures and pressures, have been optimized for AR extraction; examples include accelerated solvent extraction (ASE) [20] and supercritical fluid extraction (SCFE) using supercritical CO_2_ [23,24]. In addition, operational parameters can influence extraction efficiency, including the particle size of the raw material, temperature, and extraction duration, all of which affect the solvent’s diffusion and the solubilization of target compounds [21]. An ideal solvent for AR extraction should have a polarity similar to the solute and be safe, selective, and easy to handle [21]. In light of contemporary green chemistry principles, the choice of extraction solvents and techniques should also consider environmental sustainability, solvent toxicity, and energy efficiency [25]. Accordingly, approaches like ultrasound-assisted extraction and the use of mid-polarity, food-grade solvents such as acetone align with efforts to reduce the ecological footprint of natural product recovery. Recent advances in natural product extraction include greener strategies such as natural deep eutectic solvents (NADES), ionic liquids, and nanoparticle-assisted methods that enhance compound recovery while reducing environmental impact [26]. These techniques are increasingly explored in phenolic lipid recovery and merit consideration for future AR extraction optimization.

Specific extraction techniques can affect AR yield by manipulating these conditions. For instance, high temperatures can enhance solute diffusion and solubility but may risk the degradation of thermolabile AR homologs [27]. In this regard, extraction time also plays a critical role, with equilibrium between the solute and solvent governing the point of maximum yield [27]. Some techniques, such as Soxhlet extraction—which applies continuous reflux at solvent boiling temperature—and ultrasound-assisted extraction—which relies on cavitation-induced solvent penetration and molecular agitation—have been widely adopted for natural product isolation, including in phenolic compounds [21,28]. Despite numerous studies on AR extraction, there is still a limited understanding of how specific extraction conditions affect the AR profile and how these compositional differences translate into functional bioactivities such as cancer cell inhibition.

However, there remains a limited understanding of how extraction conditions influence the selective recovery of specific AR homologs—particularly those with distinct structural modifications—and how these variations affect bioactivity. Addressing this knowledge gap, the present study aimed to: (1) compare the efficiency of the extraction methods commonly used—maceration, Soxhlet, and ultrasound-assisted extraction—for the recovery of ARs from commercial wheat bran; (2) assess the composition of AR homologs in the resulting extracts, using targeted HPLC-DAD-MS profiling; and (3) evaluate the relationship between AR profiles and antiproliferative activity against PC-3 prostate cancer cells. By identifying extraction conditions that enhance AR yield and bioactivity, this work contributes to the development of food-derived ingredients and potential adjuvant agents for prostate cancer management.

## 2. Materials and Methods

### 2.1. Food Material and Extraction Procedure

Commercial wheat bran was purchased from a local supermarket in Bogotá, Colombia (2022 harvest). The product was labeled as stabilized, micronized wheat bran suitable for human consumption. No additional physicochemical characterization (e.g., moisture, protein, or fiber content) was performed. A single batch was used for all extraction treatments in order to ensure sample uniformity. Before extraction, the wheat bran was milled using a laboratory grinder (mesh size ~0.05 mm) to improve surface area and facilitate solvent penetration. This particle size corresponds to that of typical commercial-grade milled wheat bran and was selected to simulate realistic food processing conditions. Further micronization could enhance extraction kinetics, and this variable can be explored in future optimization studies. In all treatments, 5.00 g of ground wheat bran was used per extraction, and the solvent-to-solid ratio was standardized at 10:1 (*v*/*w*), using 50 mL of solvent for each condition. All solvents used in the extraction (*n*-hexane, acetone, and methanol) were of analytical grade with ≥99% purity (Merck, Darmstadt, Germany). Methanol and isopropanol for HPLC analysis were HPLC-grade (≥99.9% purity) (Merck, Darmstadt, Germany). Fast Blue RR reagent and standards were purchased from Sigma-Aldrich (St. Louis, MO, USA) with ≥95% purity.

The AR extraction was performed using overnight maceration, Soxhlet, and ultrasound. Eight extraction conditions were evaluated, based on variations in solvent type and extraction time: (1.) Soxhlet with acetone only (SAO); (2–4.) sequential Soxhlet-based extraction using solvents of increasing polarity, such as (2.) *n*-hexane (S-H), (3.) acetone (S-A), and (4.) methanol (S-M); (5.) overnight solvent-assisted maceration with acetone (OSAM), (6–8.) ultrasound-assisted extraction (UAE) with acetone performed at different durations, such as (6.) 10 min (UA-10), (7.) 15 min (UA-15), and (8.) 20 min (UA-20). UAE was carried using an Elmasonic P sonicator (Elma Schmidbauer GmbH, Singen, Germany) operated under the following parameters: 30 °C, 100% amplitude, and an 80 kHz frequency. A summary regarding the methods and conditions employed is presented in Table 1. After extraction, all samples were concentrated under reduced pressure and dried. Each extraction condition was performed in three replicates. The resulting extracts were stored at −20 °C until analysis.

### 2.2. Determination of Total-AR Content

Dried extracts were reconstituted in HPLC-grade methanol at 5 mg/mL, and the total-AR content was quantified using the Fast Blue RR^®^ (FBRR) colorimetric microassay. Briefly, in a 96-well plate, each well received 20 µL of 1% K_2_CO_3_, 30 µL of sample solution, and 180 µL of FBRR working reagent (1 mg/mL). Plates were incubated in the dark for 20 min, and absorbance was then measured at 480 nm using an EZ Read 800 ELISA reader (Biochrom, Cambridge, UK). All measurements were performed in three replicates. Total-AR content was expressed as micrograms of olivetol equivalent per gram dry weight (mg OE/g DW). The assay exhibited a linear response from 5–50 µg/mL olivetol (R^2^ > 0.99), with a limit of detection (LOD) and quantification (LOQ) of 2.4 µg/mL and 5.0 µg/mL, respectively.

### 2.3. Liquid Chromatography–Electrospray Ionization–Mass Spectrometry (LC-ESI-MS)

Chemical profiling was conducted using Ultra-Fast Liquid Chromatography coupled with Diode Array Detection and Electrospray Ionization Mass Spectrometry (UFLC-DAD-ESI-MS) on a Shimadzu Prominence LC2020 system (Columbia, MD, USA). Aliquots (30 µL) of each replicate per treatment prepared at 5 mg/mL were injected into a Kinetex^®^ C18 column (150 × 4.6 mm, 2.6 µm; Phenomenex Inc., Torrance, CA, USA). The column was maintained at 35 °C and the flow rate was 0.6 mL/min. The mobile phase consisted of methanol:water (8:2, *v*/*v*) (solvent A) and methanol:isopropanol (7:3, *v*/*v*) (solvent B). The gradient program was as follows: 0–2 min, 0% B; 2–27 min, linear increase to 100% B; 27–32 min, hold at 100% B; 32–34 min, return to 0% B; and 34–37 min, equilibrate at 0% B. This LC method was developed for AR separations [29]. Mass spectrometry involved the following parameters: ESI operated simultaneously in positive (+1.9 kV) and negative (−1.7 kV) ion modes across the *m*/*z* range of 150–2000, nitrogen as nebulizer (1.5 L/min) and drying (15 L/min) gas, interface and desolvation line maintained at 185 °C, and the heat block at 400 °C. The detected compounds were identified based on diagnostic interpretation of their mass spectra and UV absorption profiles, in conjunction with comparisons to published data [29]. Quantification of the detected ARs was carried out using an external standard approach utilizing LC-DAD, following the same chromatographic conditions. A calibration curve was prepared using seven (5 to 100 µg/mL) concentration points of olivetol (primary reference standard-grade phyproof^®^ reference substance, Sigma-Aldrich, St. Louis, MO, USA). The linearity was confirmed with an R^2^ value of 0.9991. Only concentrations above the LoQ (vide infra) were used for quantification. Quercetin (50 µg/mL; Sigma-Aldrich) was included as an internal standard in order to monitor instrumental performance and ensure consistency. Due to the limited commercial availability and purity of AR reference compounds, quantification was performed using olivetol equivalents. Therefore, results were expressed as micrograms of olivetol equivalents per gram of dry weight (µg OE/g DW), and relative response factors were applied to adjust peak areas accordingly. The entire analytical process, including extraction, quantification, and LC-MS analysis, was performed in triplicate. Each resulting triplicate was then injected into the LC-MS instrument in duplicate. The method’s precision was validated through intra- and inter-day analyses of olivetol, yielding relative standard deviations (RSD%) of 2.9% and 4.1%, respectively. The method’s limit of detection (LOD) and quantification (LOQ) were determined to be 1.3 µg/mL and 2.5 µg/mL, respectively. In addition, post-acquisition data processing was conducted using MZmine software v.2.53. Chromatographic baselines were corrected, and total ion current (TIC) values were exported as CSV files. Data were subsequently normalized and autoscaled. The resulting autoscaled profiles were visualized and compared across extraction treatments using stacked overlays in OriginPro 8.5, providing an intuitive visualization of AR distribution among the different extracts.

### 2.4. Antiproliferative Assay

The human prostate cancer cell line PC-3 (ATCC CRL-7934) and normal mouse fibroblast line L929 (ATCC CRL-6364) were cultured in Dulbecco’s Modified Eagle Medium (DMEM) supplemented with 10% (*v*/*v*) fetal bovine serum (FBS), 1% (*v*/*v*) penicillin, and 1% (*v*/*v*) streptomycin. Cells were maintained at 37 °C in a humidified incubator with 5% CO_2_. Antiproliferative effects of the AR-enriched extracts were assessed following a previously reported MTT-based protocol [30]. Cells were seeded at a density of 5 × 10^3^ cells per well in 96-well plates (100 µL/well) and incubated for 24 h. The medium was then replaced with serum-free DMEM containing increasing concentrations of the extracts (0.5–500 µg/mL). Each concentration was tested in triplicate. Control groups included PBS (blank), 1% (*w*/*v*) BSA in medium (negative control, 100% viability), and curcumin (0.1–100 µg/mL) as a positive control. After 48 h of treatment, 10 µL of MTT solution (5 mg/mL) was added to each well, and plates were incubated at 37 °C for 3 h under 5% CO_2_ atmosphere. The resulting formazan crystals were solubilized with DMSO (100 µL), and absorbance was recorded at 570 nm using a Varioskan LUX plate reader (Thermo Fisher Scientific, Waltham, MA, USA). The half-maximal inhibitory concentration (IC_50_) was calculated from dose–response curves, using non-linear regression, in GraphPad Prism 7.0 (GraphPad, San Diego, CA, USA).

### 2.5. Statistical Analysis

Total-AR content data were first assessed for normality using the Shapiro–Wilk test (*p* > 0.05). Differences among extraction conditions were analyzed by one-way analysis of variance (ANOVA), followed by Tukey’s post hoc test (*p* < 0.05), utilizing RStudio v1.3.1093. The LC-derived quantitative data were pre-processed (mean-centered without scaling) for sparse partial least squares discriminant analysis (sPLS-DA) and variable importance in the projection (VIP) using the Metaboanalyst 6.0 webtool [31].

## 3. Results and Discussion

Wheat bran was used to evaluate three extraction methods used for obtaining the AR-enriched extracts: Soxhlet, UAE, and overnight solvent-assisted extraction (OSA). Acetone was selected as the primary solvent due to its reported high affinity for alkylresorcinols (AR), which enhances extraction yields [29]. However, other solvents were also sequentially evaluated under Soxhlet conditions to assess their extraction efficiency and affinity for AR across the sequential fractionation. Additionally, different extraction times (10–20 min) were tested in UAE to observe changes in extraction yield. Eight treatments were compared to identify the optimal method for AR extraction from wheat bran, a matrix known to contain high AR concentrations [32]. The study also aimed to explore the relationship between each extraction method, the relative abundance and selectivity of AR homologs, and their influence on the antiproliferative activity against PC-3 prostate cancer cells.

### 3.1. Variation of Total-AR Content by Different Extraction Methods

The highest total-AR content values were observed for UAE treatments at 10, 15, and 20 min (1236.1 ± 15.0, 1257.6 ± 6.6, and 1330.8 ± 16.4 µg OE/g DW, respectively), followed by Soxhlet extraction with acetone only (1157.1 ± 9.0 µg OE/g DW) (Table 2).

These total-AR content results indicated that intensive extraction methods significantly improve AR recovery due to the strong physical forces they apply. UAE relies on cavitation, in which rapid pressure fluctuations generate microbubbles that collapse violently, promoting solvent penetration into the matrix and enhancing extraction [28]. Additionally, prolonged sonication increases temperature, which further improves extraction efficiency [21]. On the other hand, Soxhlet extraction employs heat and continuous solvent recycling, promoting a sustained and effective extraction process through constant solvent diffusion [22,33].

In contrast, OSAM yielded a lower total-AR content, 910.3 ± 3.7 µg OE/g DW (Table 2). While OSAM is the method conventionally used for extracting plant metabolites, it seems to be less efficient for AR, possibly due to slower solvent diffusion over extended periods [21]. Nonetheless, it remains a suitable option for removing compounds sensitive to heat. In this study, the relatively high AR yields obtained from Soxhlet and UAE suggest that ARs are not immediately degraded by moderate heat exposure, supporting their classification as non-thermolabile compounds [20]. However, findings from Tian et al. [34] indicate that ARs can undergo significant degradation under prolonged and intense thermal treatments, such as oven-drying or autoclaving, due to oxidation and structural breakdown of the alkyl side chains. This fact suggests that while ARs are relatively heat-stable under typical extraction conditions, their integrity may still be compromised under sustained or excessive thermal stress. In the case of UAE, although it is a milder technique, extended sonication can produce localized heat through cavitation, which may negatively impact the stability of AR homologs. Our results showed that UAE for 20 min yielded high AR content; however, based on Tian et al.’s findings [34], prolonged UAE could risk partial degradation of ARs, particularly in the presence of oxygen or light. Therefore, shorter UAE durations (e.g., 10–20 min) may offer a better balance between extraction efficiency and the preservation of AR structural integrity.

The lowest total-AR contents were observed in the serial Soxhlet extraction (*n*-hexane → acetone → methanol), with individual yields of 733.8 ± 10.1 mg OE/g DE (*n*-hexane), 230.6 ± 4.3 mg OE/g DE (acetone), and 205.6 ± 4.4 mg OE/g DE (methanol) (Table 2). These results suggest that nonpolar and moderately polar solvents exhibit some affinity for ARs due to their amphipathic nature [35]. However, the sequential solvent extraction approach appears to fractionate ARs across different solvent phases, promoting the enrichment of AR in a single extract, particularly less polar ones. In contrast, Soxhlet extraction with acetone alone provided a total-AR content that was higher than the sum of the fractions obtained from the sequential approach, confirming acetone as the most effective solvent among those tested [22].

### 3.2. Chemical Characterization of Wheat Bran Extracts by LC-MS

**Figure 1:**
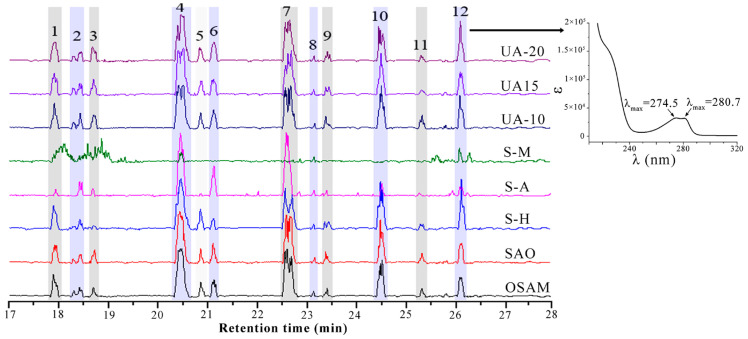
Selected LC-MS profiles, in negative ion mode, of extracts obtained by different AR-extraction methods under an AR-targeted gradient. Extraction methods: OSAM = Overnight solvent-assisted maceration with acetone (OSAM), SAO = Soxhlet with acetone only (SAO); S-H = Sequential Soxhlet-based extraction using *n*-hexane, S-A = Sequential Soxhlet-based extraction using acetone (S-A), S-M = Sequential Soxhlet-based extraction using methanol; UA-10 = Ultrasound-assisted extraction (UAE) with acetone for 10 min, UA-15 = UAE with acetone during 15 min, UA-12 = UAE with acetone during 20 min. The numbers above each signal represent the AR compounds, annotated as shown in Table 3. The plot pointing to signal 12 (C_25_) corresponds to the characteristic UV absorption spectrum of AR compounds.

The resulting enriched AR extracts were analyzed using a targeted HPLC-DAD-MS-based metabolite profiling approach, employing a specialized chromatographic system optimized for AR compounds [29]. The system used two solvent mixtures as mobile phases—phase A: methanol–water (8:2) and phase B: methanol–isopropanol (7:3)—to efficiently separate AR-type compounds across all treatments. Mass spectrometry confirmed the presence of the AR compounds by matching signals to known AR homologs. UV spectra of all putative ARs exhibited characteristic dual absorption maxima at 274–276 and 280–281 nm (Figure 1) [36].

Twelve AR homologs were annotated across all treatments after UV and mass spectral profiles were inspected (Figure 1) [37]. These homologs belonged to three structural classes of AR-type compounds: saturated, monounsaturated, and those bearing a keto substituent on the lateral chain (Table 3). 

Saturated AR homologs (C_17_–C_25_) were the most prevalent, consistent with their well-documented dominance—accounting for over 85% of total alkylresorcinols—in the kernels and bran of Poaceae species such as wheat, rye, and triticale [23,24,38,39]. In contrast, unsaturated AR homologs are rarely detected; however, C_17:1_, C_19:1_, and C_21:1_ are the homologs most frequently reported in wheat. This study also detected the less commonly reported C_21:1_, C_23:1_, and C_25:1_ homologs [40]. Their limited detection is often attributed to chromatographic sensitivity, with the variability depending on the optimal extraction conditions and the status of the plant sources [22]. Lastly, AR homologs containing a keto substituent on the lateral chain were the least abundant, and have primarily been described in wheat and rye kernels [41]. These oxo-containing derivatives were particularly found in the ARs with the most extended side chains (C_21_–C_25_) (Table 3).

### 3.3. Targeted Metabolite Profiling-Based Analysis of Wheat Bran Extracts

The AR-targeted MS dataset was pre-processed to improve peak resolution and eliminate noise and contaminants [42,43]. Quantitative analysis, expressed in olivetol equivalents (µg OE/g DW), and using LC-DAD, revealed notable differences in AR concentrations, depending on the extraction method. The quantitative profiles of twelve AR homologs (Table 4) across eight extraction treatments show an apparent variation in both total-AR content and specific homolog distribution, with respect to the saturated (e.g., C_17_ C_19_, C_21_, C_23_, and C_25_), monounsaturated (C_19:1_, C_21:1_, C_23:1_, and C_25:1_), and keto-substituted forms (C_21:Oxo_, C_23:Oxo_, and C_25:Oxo_), depending on the extraction method.

The ultrasound-assisted extractions at 80 kHz (UA-10, UA-15, and UA-20) consistently yielded the highest concentrations of total ARs. Notably, UA-20 produced the most abundant extract, one with the highest concentrations of C19 (411.5 ± 14.0 µg/g) and C_21_ (390.8 ± 10.5 µg/g), and substantial levels of C_25_ (108.1 ± 4.1 µg/g). This outcome confirms the effectiveness of high-frequency ultrasound in enhancing solvent penetration and alkylresorcinol (AR) release through cavitation-driven mechanisms. Notably, longer extraction times further amplify this effect. In contrast, when increasingly high-power ultrasound-assisted extraction systems (>250 W) are used, shorter extraction durations (typically <150 s) are generally sufficient [34,44].

Among the Soxhlet-based methods, SAO (Soxhlet with acetone only) yielded high AR levels across most homologs, particularly saturated ARs such as C_21_ (404.0 ± 14.5 µg/g), C_19_ (325.0 ± 16.8 µg/g), and C_23_ (146.6 ± 7.1 µg/g), suggesting that acetone is a highly effective solvent for AR recovery under thermal recirculation conditions. In contrast, serial Soxhlet extractions (S-A, S-H, and S-M) generally underperformed, likely due to compound partitioning across multiple fractions and reduced selectivity for individual homologs. The OSAM (overnight solvent-assisted extraction) showed moderate AR concentrations but remained notably lower than UAE and SAO, particularly for C_21_ and C_19_. This observation is consistent with the slow passive diffusion associated with maceration, which may be less effective in fully mobilizing ARs from the bran matrix. Interestingly, keto-substituted ARs (e.g., C_21:Oxo_, C_23:Oxo_, and C_25:Oxo_) were recovered in significant quantities under UA and SAO conditions but were considerably lower in the serial Soxhlet methods, suggesting that these homologs may be more labile or less extractable under extended solvent cycling. The data indicate that ultrasound-assisted extraction at 20 min (UA-20) and Soxhlet with acetone only (SAO) were the most effective approaches in extracting a broad spectrum of AR homologs, both in terms of yield and diversity. In this regard, targeted LC-MS metabolite profiling is reliable for evaluating extraction efficiency and homolog selectivity.

**Figure 2:**
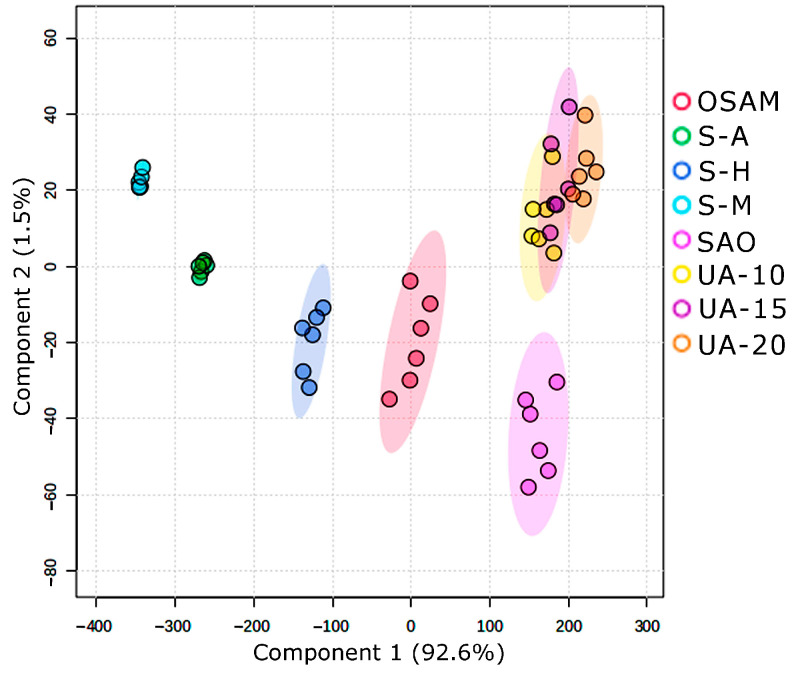
Scores plot from sparse partial least squares discriminant analysis (sPLS-DA) based on LC-MS data (*n* = 6) for alkylresorcinol (AR) homologs extracted under different conditions. Extraction conditions include overnight solvent-assisted maceration (OSAM), Soxhlet-acetone (S-A), Soxhlet-*n*-hexane (S-H), Soxhlet-methanol (S-M), Soxhlet with acetone only (SAO), and ultrasound-assisted extraction at 10, 15, and 20 min (UA-10, UA-15, and UA-20).

To better visualize the distribution of the different extraction methods and identify patterns that reveal the conditions that most strongly influenced sample discrimination, the quantitative data were analyzed using sparse partial least squares discriminant analysis (sPLS-DA) with mean-centering pre-processing and without scaling. [45]. The resulting sPLS-DA-derived scores plot (Figure 2) reveals a clear and structured separation of the samples according to the different extraction methods applied for AR recovery.

Component 1, which accounts for 92.6% of the total variance, is the primary driver of discrimination, while Component 2 contributes only 1.5%, offering some additional resolution. Samples obtained through ultrasound-assisted extraction (UA) form distinct clusters toward the right side of the plot, with UA-10, UA-15, and UA-20 each grouping separately but closely, suggesting that extraction time exerts a subtle influence on the AR profile. These clusters are well separated from those corresponding to the conventional Soxhlet-based methods (S-A, S-H, and S-M) located on the far left (Figure 2). S-H and S-A appear tightly grouped, indicating low intra-group variability and similar chemical profiles, likely reflecting limited extraction efficiency under those conditions.

Between these two extremes, solvent-assisted overnight maceration (OSAM) and Soxhlet with optimized acetone (SAO) cluster toward the center-right of the plot. Their intermediate position implies a moderate capacity for AR extraction, distinguishing them from the highly efficient UA treatments and the less effective Soxhlet-*n*-hexane or -acetone approaches. The overall distribution of points and tight grouping within treatments underscore the robustness of the sPLS-DA model in discriminating between extraction strategies. The clear separation observed along Component 1 emphasizes that the extraction method is the dominant factor influencing the AR profile, with ultrasound-assisted techniques producing the most chemically distinct and variable extracts. This pattern supports the effectiveness of high-frequency ultrasound in modifying extraction behavior, likely through cavitation-enhanced solvent penetration and compound release.

**Figure 3:**
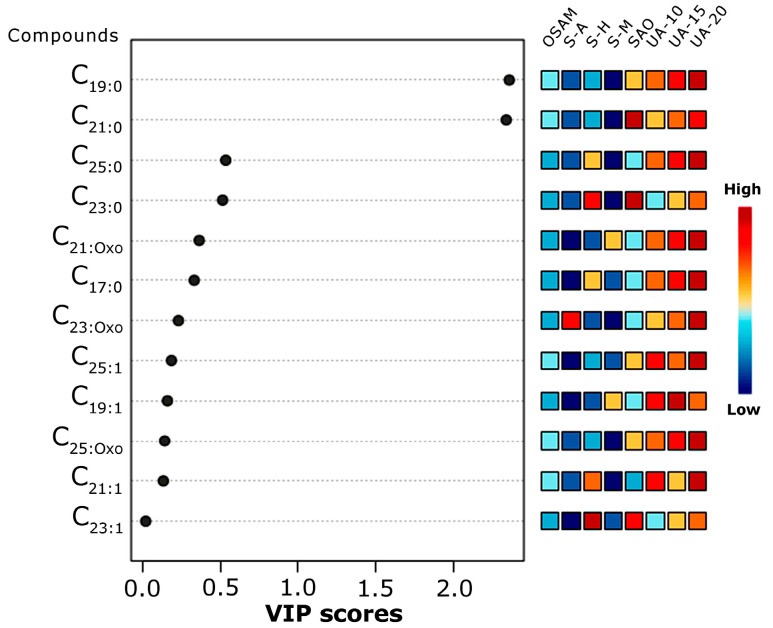
Variable importance in projection (VIP) scores from sPLS-DA of AR homologs across extraction methods. AR homologs are labeled according to chain length and functional class (e.g., C_21:Oxo_, C_25:1_). Data were mean-centered prior to sPLS-DA, without further transformation. The plot displays the VIP scores of individual AR homologs, indicating their relative importance in discriminating against extraction techniques. The adjacent heatmap represents the influence of each AR across the extraction methods (dark red: high influence; dark blue: low influence). Extraction conditions include overnight solvent-assisted maceration (OSAM), Soxhlet-acetone (S-A), Soxhlet-*n*-hexane (S-H), Soxhlet-methanol (S-M), Soxhlet with acetone only (SAO), and ultrasound-assisted extraction at 10, 15, and 20 min (UA-10, UA-15, and UA-20).

The influence of the extraction method on the recovery of individual AR homologs was directly assessed by generating a variable importance in projection (VIP) plot (Figure 3) from the sPLS-DA model using mean-centered quantitative data. This plot highlights the ARs that most strongly contribute to the discrimination among extraction conditions.

The VIP scores indicate that C_19_ and C_21_ are the most influential variables in differentiating extraction methods, both displaying VIP values above the commonly accepted threshold of 1.0 [46]. This finding suggests these two AR homologs are particularly sensitive to extraction conditions and may be key markers for evaluating extraction efficiency. Additional homologs, such as C_25_, C_23_, C_21:Oxo_, and C_17_, also showed moderate importance, with VIP scores approaching 0.5–1.0, indicating a secondary but still relevant contribution to group separation (Figure 3).

The heatmap accompanying the VIP scores provides further insight into the relative influence of each extraction method on AR removal. A clear trend is observed: ultrasound-assisted extraction at higher times (UA-15 and UA-20) consistently leads to higher levels of the most influential ARs (e.g., C_19_, C_21_, and C_25_), as indicated by the red-to-orange color gradient. In contrast, Soxhlet and overnight solvent-assisted extraction (S-A, S-H, S-M, and SAO) are associated with lower AR levels (blue shades), reinforcing their comparatively lower extraction performance. The VIP analysis underscores the selectivity and efficiency of high-frequency ultrasound-assisted extraction in enhancing the recovery of specific AR homologs, particularly C_19_ and C_21_, which may be critical indicators for optimizing extraction protocols.

### 3.4. Antiproliferative Activity of Wheat Bran Extract Against the PC-3 Cell Line

The cytotoxic potential of the AR-enriched extracts obtained from various extraction protocols was ultimately evaluated against PC-3 cancer cells by determining the IC_50_ values (concentration required to inhibit 50% of cell proliferation). The results revealed marked differences in antiproliferative activity across extraction conditions (Table 5), with IC_50_ values ranging from 13.3 µg/mL for UA-20 to 55.6 µg/mL for S-M. These findings indicate that the extraction technique significantly influences the bioactivity of the resulting AR mixtures.

Among all tested conditions, the ultrasound-assisted extraction at 20 min (UA-20) yielded the most potent extract, the one with the lowest IC_50_ value, 13.3 µg/mL (95% CI: 11.9–14.8 µg/mL), followed closely by determinations for UA-15 (14.3 µg/mL) and UA-10 (17.5 µg/mL). These findings highlight the effectiveness of high-frequency ultrasound in enhancing the biological activity of the AR-rich extracts, possibly by improving the extraction of specific bioactive homologs or preserving their functional integrity. In contrast, extracts obtained from Soxhlet-acetone (S-A) and Soxhlet-methanol (S-M) exhibited significantly weaker cytotoxicity, with IC_50_ values of 30.5 µg/mL and 55.6 µg/mL, respectively. This reduced activity likely reflects the sequential nature of the extraction, as a prior Soxhlet extraction with *n*-hexane removed a substantial portion of ARs, resulting in less efficient recoveries in subsequent solvent phases. Notably, the *n*-hexane fraction (S-H) showed moderate antiproliferative activity (IC_50_ = 22.6 µg/mL), supporting a finding that key bioactive AR homologs had already been depleted before acetone and methanol extractions were performed. The overnight solvent-assisted maceration (OSAM) and Soxhlet with acetone only (SAO) methods performed moderately well, yielding IC_50_ values of 20.3 µg/mL and 18.3 µg/mL, respectively. These data suggest a strong link between the extraction method, AR profile, and antiproliferative potency. In particular, ultrasound-assisted extraction—especially at 15–20 min—appears to optimize the recovery of AR homologs with enhanced cytotoxic potential, positioning it as a promising approach for producing biologically active AR-rich extracts.

A clear trend emerges when linking AR profile and cytotoxic potential, particularly when observed by multivariate analysis through the VIP plot (Figure 3), which reinforces the differential influence of AR homologs on the antiproliferative response of PC-3 cells. In agreement with the variable importance highlighted in the sPLS-DA model, the ultrasound-assisted extracts (UA-10, UA-15, and UA-20), which showed the highest antiproliferative activity (IC_50_: 17.5–13.3 µg/mL), were characterized by consistently high levels of C_21_, C_19_, and, especially, oxygenated AR homologs such as C_21:Oxo_, C_23:Oxo_, and C_25:Oxo_. For instance, UA-20 had the highest quantities of C_21:Oxo_ (52.0 µg/g) and C_23:Oxo_ (69.3 µg/g), coinciding with its antiproliferative effect, which was the most substantial of the tested substances (IC_50_ = 13.3 µg/mL). These keto-containing ARs have been previously linked to increased cytotoxicity against prostate cancer cells, including the PC-3 line, due to the ability of these ARs to modulate apoptotic pathways and mitochondrial function [47]. In contrast, extracts with weaker bioactivity, such as S-M (IC_50_ = 55.6 µg/mL) and S-A (30.5 µg/mL), contained lower concentrations of both total ARs and oxygenated homologs, while saturated ARs (e.g., C_17_, C_19_, and C_21_) remained relatively modest. These observations suggest that total-AR content alone is not a sufficient predictor of activity—instead, the presence and proportion of specific bioactive AR subclasses, particularly keto-substituted homologs, may be more relevant.

Interestingly, S-H, although intermediate in total-AR content, presented moderate activity (IC_50_ = 22.6 µg/mL), likely due to higher levels of C_19_ and C_21_ but a relatively low content of oxygenated forms. In contrast, OSAM and SAO, with balanced profiles rich in both saturated and oxygenated ARs (e.g., C_23:Oxo_ ~45–46 µg/g), also showed strong antiproliferative activity (IC_50_ = 20.3 and 18.3 µg/mL, respectively). This trend suggests that saturated ARs may be less effective in promoting antiproliferative activity. One possible explanation is the influence of side-chain length, as shorter-chain ARs appear to enhance cytotoxicity, potentially due to better cellular absorption compared to their longer-chain counterparts [17]. These findings collectively suggest that oxygenated ARs (C_21:Oxo_, C_23:Oxo_, and C_25:Oxo_) may be closely associated with increased antiproliferative potential, likely due to structural features that enhance cellular uptake or bioactivity. Our observation that oxygenated ARs show stronger antiproliferative effects aligns with previous findings by Fu et al., who reported enhanced apoptosis and p53 activation in colon cancer cells treated with structurally modified ARs [7]. Similarly, El-Shabasy and Farag emphasized the relevance of specific AR subclasses for therapeutic development, highlighting the value of compositional profiling alongside cytotoxic evaluation [18]. This structure–activity relationship highlights the importance of the optimization of extraction methods for yield and selective enrichment of bioactive AR homologs relevant to food-related product development and derived applications.

Among the extraction methods, acetone-based Soxhlet and ultrasound-assisted extraction (UAE) clearly outperformed others in terms of both AR yield and cytotoxic potential, aligning with previous reports that emphasize the effectiveness of low-to-intermediate-polarity solvents in extracting phenolics and ARs from cereal matrices [20,48]. The elevated bioactivity observed in extracts under UA-20 and SAO conditions may be attributed to the tendency of these conditions to concentrate oxygenated AR homologs, which have been associated with apoptotic activity and mitochondrial disruption in cancer cells. The high lipophilicity of ARs (LogP ≈ 7–10), driven by their long alkyl side chains, enhances passive diffusion through lipid membranes, and may improve their cellular uptake and retention in tumor cells. This property is especially relevant for keto-substituted ARs, which were recovered in higher proportions under UAE and SAO conditions and may be more readily internalized due to their combined lipophilic and polar characteristics. However, despite its efficiency, UAE poses limitations that warrant attention—extended sonication can generate localized heating through cavitation, which might compromise the stability of thermolabile AR homologs, particularly in oxygen-rich environments. Thus, optimization of UAE parameters, including time, temperature, and sonication frequency, is essential to balance extraction efficiency with structural preservation. Future studies should also explore how variations in AR side-chain polarity and unsaturation influence cellular uptake dynamics and intracellular targets.

While our study focused on alkylresorcinols (ARs) extracted from wheat bran, it is worth noting that other cereals, such as rye, barley, and triticale, also contain ARs with varying homolog profiles. However, according to the systematic review by Kruk et al. [17], the biological activity of ARs—particularly anticancer or cytotoxic properties—has been predominantly characterized in wheat-derived ARs. There remains a scarcity of comparative data for ARs from other cereals in terms of both structural composition and functional effects in biological models. Future work should aim to explore the bioactivity of cereal-specific AR profiles to fully understand their functional diversity and therapeutic potential.

## 4. Conclusions

This study systematically investigated multiple extraction strategies employed to remove ARs from wheat bran, and the profiles of the resulting extracts were analyzed using a targeted HPLC-DAD-MS metabolite profiling approach. Twelve AR homologs were identified, including saturated, monounsaturated, and keto-substituted variants, predominantly featuring side chains ranging from C_17_ to C_25_—typical of Poaceae species. Among the evaluated methods, overnight solvent-assisted maceration (OSAM) and Soxhlet with acetone only (SAO) extractions yielded the highest total-AR content, particularly enriching key bioactive homologs. In contrast, conventional Soxhlet extraction without pre-treatment was the least effective, while ultrasound-assisted methods showed different but improved performance, regardless of the extraction time. In this regard, ultrasound-assisted extraction at 20 min (UA-20) yielded 30% more total ARs, compared to Soxhlet extraction. Further cost–benefit analysis could validate the industrial applicability of this approach. Multivariate statistical analyses, including supervised approaches such as sPLS-DA, clarified the AR distribution patterns and group separation. These analyses highlighted key discriminatory metabolites—most notably C_25_, C_19:1_, and C_23:Oxo_—as markers differentiating the extraction techniques. In this regard, biological assays showed that extracts with relatively higher levels of keto-substituted ARs—particularly those from the UAE and OSAM methods—exhibited stronger antiproliferative activity against PC-3 prostate cancer cells. In contrast, extracts enriched in saturated homologs, such as C_17_ and C_19_, were less effective, suggesting a potential structure–activity relationship influenced by both the degree of unsaturation and side-chain length. These findings support previous evidence that shorter and oxygenated AR homologs may be more bioavailable and biologically active. This study reinforces the concept that extraction conditions not only influence the total yield of AR from wheat bran, but also determine the structural composition of the recovered AR homologs, which in turn impacts the antiproliferative potential of these homologs. By comparing different extraction strategies, we demonstrate that modest enrichment of oxygenated ARs, as in, e.g., keto-substituted homologs, enhances bioactivity against PC-3 cancer cells. These findings emphasize the importance of tuning extraction parameters to optimize both chemical composition and functional outcomes. The results lay a solid foundation for developing AR-enriched food-derived products and for future studies exploring their mechanisms of bioactivity and health-promoting potential. Future studies should also validate the findings across different wheat bran sources, considering regional and cultivar-based variability. Moreover, the incorporation of AR-enriched extracts into model food systems will be essential to assess the functional stability, sensory compatibility, and real-world health benefits of these homologs.

## Figures and Tables

**Table 1:** Summary of extraction conditions, including solvent type, extraction mode, duration, and acronyms used throughout the study.

Extraction Method	Solvent	Type	Duration	Acronym
Soxhlet	acetone	Continuous Hot	~6 h	SAO
Soxhlet (sequential)	*n*-hexane	Sequential	~6 h each	S-H
Soxhlet (sequential)	acetone	Sequential	~6 h each	S-A
Soxhlet (sequential)	methanol	Sequential	~6 h each	S-M
Overnight maceration	acetone	Static	24 h	OSAM
Ultrasound-assisted	acetone	Ultrasound	10 min	UA-10
Ultrasound-assisted	acetone	Ultrasound	15 min	UA-15
Ultrasound-assisted	acetone	Ultrasound	20 min	UA-20

**Table 2:** Yields and total alkylresorcinol (AR) contents obtained from the test extraction methods.

Extraction Method		Yield (mg DE/g PM) ^a^	Total AR (µg OE/g DE) ^b^
Overnight solvent-assisted maceration (OSAM) ^c^		40.2 ± 2.5 ^C^	910.3 ± 3.7 ^B^
Ultrasound-assisted ^c^	10 min	30.0 ± 3.2 ^DE^	1236.1 ± 15.0 ^A^
	15 min	34.0 ± 0.2 ^D^	1257.6 ± 6.6 ^A^
	20 min	29.1 ± 1.3 ^EF^	1330.8 ± 16.4 ^A^
Soxhlet with acetone only (SAO) ^c^		48.5 ± 1.8 ^B^	1157.1 ± 9.0 ^A^
Soxhlet	*n*-hexane	26.2 ± 1.6 ^EF^	733.8 ± 10.1 ^C^
	acetone	25.0 ± 4.1 ^EF^	230.6 ± 4.3 ^D^
	methanol	59.3 ± 2.1 ^A^	205.6 ± 4.4 ^D^

All values are the means of three experimental replicates, along with their respective standard deviations (± value). Superscript capital letters indicate significant differences according to the Tukey test (*p* = 0.05). ^a^ Extraction yield: Amount of dry extract, expressed as mg dry extract/g plant material. ^b^ Total-AR content, expressed as mg olivetol equivalent/g dry extract (mg OE/g DE). ^c^ Acetone is the extractant used in these extraction methods.

**Table 3:** Annotation of 5-*n*-alkylresorcinols homologs identified by HPLC-MS (negative ion mode, extracted from wheat bran using different extraction methods).

No	Retention Time (min)	[M-H]^−^ (*m*/*z*)	Molecular Formula	Homologs	Name
1	17.9	347	C_23_H_40_O_2_	C_17_	5-*n*-heptadecylresorcinol
2	18.2	373	C_25_H_42_O_2_	C_19:1_	5-*n*-nonadecenylresorcinol
3	18.5	417	C_27_H_46_O_3_	C_21:Oxo_	5-*n*-oxoheneicosanylresorcinol
4	20.4	375	C_25_H_44_O_2_	C_19_	5-*n*-nonadecanylresorcinol
5	21.7	401	C_27_H_46_O_2_	C_21:1_	5-*n*-heneicosenylresorcinol
6	21.1	445	C_29_H_50_O_3_	C_23:Oxo_	5-*n*-oxotricosanylresorcinol
7	22.6	403	C_27_H_48_O_2_	C_21_	5-*n*-heneicosylresorcinol
8	23.1	473	C_31_H_54_O_3_	C_25:Oxo_	5-*n*-oxopentacosanylresorcinol
9	23.4	429	C_27_H_48_O_2_	C_23:1_	5-*n*-tricosenylresorcinol
10	24.4	431	C_29_H_52_O_2_	C_23_	5-*n*-tricosylresorcinol
11	25.3	457	C_29_H_52_O_2_	C_25:1_	5-*n*-pentacosenylresorcinol
12	26.0	459	C_31_H_56_O_2_	C_25_	5-*n*-pentacosylresorcinol

**Table 4:** Quantitative profiles of alkylresorcinol (AR) homologs in wheat bran extracts obtained using different extraction methods.

Sample	AR Quantitative Data ^a^											
	C_17_	C_19:1_	C_21:Oxo_	C_19_	C_21:1_	C_23:Oxo_	C_21_	C_25:Oxo_	C_23:1_	C_23_	C_25:1_	C_25_
**OSAM**	43.5 ± 1.6	31.8 ± 1.3	25.3 ± 1.2	236.7 ± 18.9	29.3 ± 1.6	45.1 ± 1.9	271.2 ± 13.9	18.0 ± 0.9	9.0 ± 0.5	100 ± 7.6	19.0 ± 1	71.4 ± 5.4
**S-H**	49.3 ± 2.4	20.3 ± 0.9	3.7 ± 0.2	205.3 ± 12.8	31.5 ± 1.5	5.6 ± 0.3	103.7 ± 7.2	6.9 ± 0.4	32.8 ± 1.8	145.6 ± 8.3	16.8 ± 1.2	96.2 ± 8.7
**S-A**	15.3 ± 0.3	23.8 ± 0.2	32.7 ± 0.1	80.8 ± 3.9	3.4 ± 0.1	62.4 ± 3.1	78.5 ± 3.1	17.1 ± 0.2	5.3 ± 0.2	14.4 ± 0.5	4.8 ± 0.2	15.5 ± 0.4
**S-M**	10.3 ± 1.2	14.5 ± 1.3	7.6 ± 1.1	23.0 ± 1.8	3.1 ± 0.2	3.6 ± 0.1	17.1 ± 0.7	2.2 ± 0.3	3.6 ± 0.3	5.0 ± 0.4	1.9 ± 0.2	6.3 ± 0.5
**SAO**	47.5 ± 2.3	32.1 ± 0.7	29.9 ± 1.3	325.0 ± 16.8	27.6 ± 1.3	45.5 ± 2.5	404.0 ± 14.5	21.7 ± 0.7	14.8 ± 0.7	146.6 ± 7.1	19.2 ± 1.5	81.8 ± 3.6
**UA-10**	59.4 ± 3.4	36.5 ± 2.1	48.2 ± 2.2	362.0 ± 13.5	32.7 ± 0.9	54.2 ± 1.5	370.9 ± 7.5	23.0 ± 1.1	12.2 ± 0.5	115.2 ± 9.4	30.4 ± 1.4	100.5 ± 5.9
**UA-15**	60.0 ± 3.0	37.9 ± 2.6	48.9 ± 3.1	386.9 ± 19.1	31.5 ± 1.7	59.7 ± 1.9	372.7 ± 16.5	24.2 ± 1.2	13.0 ± 1.1	118.5 ± 9.2	30.1 ± 0.9	103.0 ± 4.2
**UA-20**	67.1 ± 3.6	34.7 ± 2.7	52.0 ± 2.2	411.5 ± 14.0	33.6 ± 1.3	69.3 ± 2.5	390.8 ± 10.5	25.3 ± 0.9	13.6 ± 0.5	125.9 ± 3.6	31.0 ± 1.5	108.1 ± 4.1

^a^ Contents (micrograms olivetol equivalent per gram dry weight, i.e., µg OE/g DW) of twelve identified alkylresorcinol (AR) homologs—ranging from C_17_ to C_25_, with saturated, unsaturated, and oxo-functionalized side chains—in wheat bran extracts obtained by various methods: OSAM (overnight solvent-assisted maceration), S-A (standard Soxhlet-acetone), S-H (standard Soxhlet-*n*-hexane), S-M (standard Soxhlet-methanol), SAO (Soxhlet with acetone only), and UA-10/15/20 (ultrasound-assisted at 10, 15, and 20 min). Values are presented as mean ± standard deviation (*n* = 3), expressed as olivetol equivalents per gram of dry weight (µg OE/g DW).

**Table 5:** Cytotoxic activity of AR-enriched extracts.

Sample ^a^	OSAM	S-H	S-A	S-M	SAO	UA-10	UA-15	UA-20
IC_50_ (µg/mL) ^b^	20.3	22.6	30.5	55.6	18.3	17.5	14.3	13.3
CI (95%) ^c^	21.2–19.3	23.8–21.1	32.1–28.8	56.9–54.2	19.8–17.4	18.5–16.6	15.2–13.5	14.8–11.9

^a^ OSAM (overnight solvent-assisted maceration), S-A (standard Soxhlet-acetone), S-H (standard Soxhlet-*n*-hexane), S-M (standard Soxhlet-methanol), SAO (Soxhlet with acetone only), and UA-10/15/20 (ultrasound-assisted at 10, 15, and 20 min). ^b^ IC_50_ = half-maximal inhibitory concentration; ^c^ Confidence interval at 95% confidence.

## Data Availability

The original contributions presented in this study are included in the article. Further inquiries can be directed to the corresponding author.

## Abbreviations

The following abbreviations are used in this manuscript:

AR	Alkylresorcinol
HPLC	High-performance liquid chromatography
MS	Mass spectrometry
ESI	Electrospray
DAD	Diode array detector
OSAM	Overnight solvent-assisted maceration
SAO	Soxhlet with acetone only
UAE	Ultrasound-assisted extraction
SPFE	Supercritical fluid extraction
ASE	Accelerated solvent extraction
sPLS-DA	Sparse partial least squares discriminant analysis
VIP	Variable importance in the projection
TIC	Total ion current
